# Exploring the effects of mental health on bonding and caregiving among pregnant and postpartum persons with likely depression and/or PTSD in South Africa: A qualitative analysis

**DOI:** 10.21203/rs.3.rs-5041479/v1

**Published:** 2024-10-24

**Authors:** Lauren Gulbicki, Madison Fertig, Jennifer Githaiga, Linda Gwangqa, Katherine Kabel, Jane Lee, Lucia Knight, Conall O’Cleirigh, Christina Psaros, Amelia Stanton

**Affiliations:** Massachusetts General Hospital; Boston University; University of Cape Town; University of Cape Town; Boston University; Boston University; University of Cape Town; Massachusetts General Hospital; Massachusetts General Hospital; Boston University

**Keywords:** Mental health, pregnancy, caregiving, HIV prevention

## Abstract

Perinatal mental health disorders place a particularly high public health burden on South Africa (SA) via negative health outcomes for the birthing parent and adverse health outcomes for infants (e.g., low birth weight, preterm deliveries, malnourishment) as well as emotional and behavioral problems in children. Depression, posttraumatic stress disorder (PTSD), and other mental health disorders may also compromise engagement in HIV prevention behaviors during the perinatal period, when HIV acquisition risk increases. This is particularly important in SA, where almost a quarter of women between ages 15 to 49 have HIV. There is little research exploring the anticipated impacts mental health symptoms have on one’s ability to emotionally connect or caregive after delivery; this critical information will enable providers to support women and their mental health during the transition from pregnancy to postpartum. HIV-negative pregnant persons were recruited from an antenatal clinic in Cape Town as a part of a larger study investigating mental health barriers to pre-exposure prophylaxis (PrEP) uptake during pregnancy. Participants qualified for an in-depth interview based on elevated symptoms of depression and/or PTSD. The interviews explored the likely impact of their mental health symptoms on their baby’s wellbeing, their ability to bond with their baby, and their ability to meet their baby’s needs. Following the principles of thematic analysis, we identified three main themes that described these relationships: (1) a strong perceived connection between maternal mental health and baby’s wellbeing; (2) perceived strains on bonding with the baby; and (3) negative impact of mental health on likelihood of completing parenting tasks. This study will inform future mental health programming to prepare pregnant persons with mental health symptoms for a successful postpartum period with respect to bonding and caring for their infant.

## Introduction

Perinatal mental health disorders, occurring during pregnancy and the first twelve months of the postpartum period, place a high public health burden on South Africa [1]. In general, prevalence rates of perinatal mental health disorders are disproportionately higher in low- and middle-income countries (LMICs) than in high-income countries [2]. In South Africa (SA), documented peripartum depression rates fall between 27%–39%, comparable to rates in other LMICs [3, 4]. Anxiety disorders are also common in SA, affecting between 15%–23% of perinatal cisgender women, with posttraumatic stress disorder (PTSD) affecting approximately 11% of this population [3, 5]. In LMICs, factors like having a history of mental health disorders outside of pregnancy, experiencing food insecurity or financial stress, exposure to childhood trauma and/or other forms of adversity, and having an unplanned or unwanted pregnancy can exacerbate risk of peripartum mental health disorders [5, 6].

Research conducted in SA has suggested that overlapping epidemics of intimate partner and gender-based violence, HIV, and structural inequalities synergistically exacerbate mental health symptoms during pregnancy and postpartum [7]. One of the strongest predictors of PTSD is intimate partner violence (IPV) [8], a form of gender-based violence defined as any behavior within an intimate partnership that causes psychological, physical, or sexual harm. In SA, IPV rates are high at approximately 31% among married and cohabiting women [9]; over half of South African women who have experienced IPV meet criteria for severe PTSD, and up to 66% meet for severe clinical depression [9]. In SA and other LMICs with high HIV prevalence rates, the HIV epidemic is important context for understanding IPV and associated mental health disparities. Worldwide, people living with HIV have recurring rates of depression and anxiety that are two to four times higher [10] than people who do not have HIV. Moreover, financial stress or the lack of sufficient resources are associated with depression [3]; these stressors and related structural challenges are common in SA.

Depression and PTSD during pregnancy may compromise the health of the birthing parent and the baby. Symptoms of perinatal depression can impair wellbeing through a loss of interest in activities, including caring for oneself or for the baby, as well as through thoughts and behaviors that lead to self-harm, or, in very rare cases, harm to the baby, or suicide [11]. Among pregnant persons in Ethiopia, antenatal depression was also associated with low antenatal care service utilization, which supports similar findings from high-income countries [12, 13]. Unique to PTSD, symptoms including hypervigilance or readily distracted attention can hinder one’s ability to caregive [14, 15]. In addition to these behavioral impairments, PTSD can also have lasting biological impacts on the infant. In a South African cohort, maternal PTSD was found to be associated with impaired neurodevelopment in the infant [16]. Additionally, common symptoms of perinatal mental health disorders are associated with poor nutrition, diminished capacity to breastfeed or nourish the baby, and increased use of substances, including tobacco and alcohol [5], which contribute to preterm deliveries, low birth weights, infant malnourishment, and neonatal abstinence syndrome (i.e., when substances are used) [17, 18]. Considering this critical developmental period, individuals exposed to mental health-related stressors *in utero* are at risk for physical, cognitive, and behavioral complications later in childhood [19].

Considerably less research has focused on the degree to which perinatal mental health disorders may also impair the maternal-child emotional relationship in SA and other LMICs. In general, mothers with depression have demonstrated decreased closeness, emotional involvement or attunement, and security in their relationships with their babies [19]. One SA-based study explored the connection between maternal mental health and the strength of the maternal-infant relationship. Mothers living with depression had significantly poorer sensitivity (characterized by responsiveness, acceptance, and warmth, in addition to actual sensitivity) when engaging with their infants [20].

There is a growing body of research on the longterm effects of perinatal mental health conditions on a child, though most of this research has been conducted in high-income countries. One study conducted in Japan found that, in the context of parental psychopathology, feelings of anger, lack of affection, and rejection towards infants, led to childhood emotional and behavioral problems [21]. Given current understanding of developmental psychology and the negative impact of adverse childhood events on adult outcomes [22], these emotional and behavioral challenges can have lifelong impacts [23], especially in contexts with limited resources and significant public health challenges. However, there is a lack of qualitative accounts from mothers exploring the anticipated impacts of their mental health symptoms on their ability to emotionally connect or caregive after delivery, and there is a disproportionate lack of research in this area coming from LMICs.

Therefore, the current study explored pregnant persons’ perceptions of the impact of their current mental health challenges on future caregiving and bonding with their infants. Individual, qualitative interviews explored perceived connections between maternal mental health and child wellbeing, bonding, and ability to engage in parenting tasks in the future. These findings will help guide future tailored interventions aimed at reducing the burden of depression and PTSD during the perinatal period, thereby improving the wellbeing of mothers and their babies in SA.

## Methods

### Parent study procedures

Participants were recruited from an antenatal clinic outside of Cape Town as part of a larger, mixed-methods study that investigated mental health barriers to pre-exposure prophylaxis (PrEP) uptake and adherence during pregnancy, as well as engagement in healthcare and ability to care for their baby. Study research assistants approached potential patients while they waited in line for care at the antenatal clinic. Those who expressed interest in participating were invited to speak with a research assistant to be screened for eligibility.

Eligible participants met the following criteria: (1) aged 18 years or older; (2) pregnant (all gestational ages permitted) and presenting for antenatal care; (3) not currently on PrEP and no history of PrEP use; and (4) HIV-negative per medical chart review.

Those who were eligible and wished to participate then completed the informed consent process and a baseline survey, which assessed mental health, relationships and social support, HIV knowledge, PrEP optimism, and likelihood of initiating PrEP during pregnancy. In the parent study, 110 participants completed this baseline survey, from which 44 participants qualified for a qualitative interview based on elevated symptoms of depression and/or PTSD.

Elevated symptoms of depression were defined by a score of ≥ 23 on the South Africa Depression Scale (SADS) [24] or ≥ 10 on the Edinburgh Postnatal Depression Scale (EPDS) [25]. The SADS is a 16-item survey developed to detect clinical depression among South Africans who speak isiXhosa. The survey inquires about local idioms of distress specific to isiXhosa culture, including “isingqala” (meaning deep sorrow). Total scores range from 0–48, with high scores indicating more severe depression. The EPDS, comprised of 10 items, was developed to screen for depression among postpartum women but has also been used extensively among pregnant persons, including in SA-based studies [26, 27]. Scores range from 0–30, with higher scores indicating increased symptom severity.

Elevated PTSD symtpoms was defined as a score ≥ 31 on the PTSD Checklist for the Diagnostic and Statistical Manual of Mental Disorders Fifth Edition (DSM-5) (PCL-5) [28]. The PCL-5 was developed alongside the DSM-5 PTSD symptom criteria to assess PTSD symptom severity. In this self-report measure, scores range from 0–80, and higher scores represent increased symptom severity.

Participants were asked to partake in two interviews, one during pregnancy and one during the postpartum period, to capture anticipated psychological barriers and facilitators to PrEP use during pregnancy and to follow up on changes in mental health symptoms and associated impacts on PrEP use postpartum. Of the 44 participants who qualified for an interview from the parent survey, 10 completed a pregnancy interview only, six completed a postpartum interview only, and seven participants completed both a baseline pregnancy interview; there were a total of 23 participants with 30 interviews included in the current analysis.

The semi-structured interview guides (Additional file 1), developed based on the principles of Huberman and Miles [29], were approved by the Human Research Ethics Committee of the Faculty of Health Sciences at the University of Cape Town, South Africa [HREC REF Number: 231/2021]. Probes varied slightly based on the population (pregnant or postpartum) and explored participants’ mental health symptoms, anticipated barriers and facilitators to PrEP uptake and persistence given those symptoms, and the ways in which a psychosocial intervention during pregnancy/postpartum could support PrEP initiation and persistence in the context of mental health challenges. Interviews were conducted by trained, bilingual (English and isiXhosa) research assistants at the clinic or over the phone depending on participants’ preferences.

### Analysis

Interviews were audio recorded, transcribed, and translated from isiXhosa to English by a member of our South African team (LG). Qualitative analyses followed the principles of thematic analysis [30] and were informed by grounded theory [31]. Two transcripts were open-coded by study authors (LRG, MF). The team members then met to discuss codes and organized them into themes and sub-themes. Another two transcripts were open coded to further refine the codebook. Team members once again met to discuss. Then four transcripts were double coded (by LRG, MF, JL, KK) using the Dedoose software [32]. Team members met to discuss and resolve any discrepancies in code definitions and coding conventions, and if discrepancies were not resolved by the coders, they were discussed with the PI (AMS) and the South Africa-based team (JNG, LG). Afterwards, the remaining transcripts were independently coded by team members (LRG, MF, JL, KK) using the refined codebook and Dedoose software. Once all interviews were coded, authors LRG and MF met to discuss final themes and extract exemplary quotes. These exemplary quotes were then brought to the full team to discuss relevant cultural interpretations and ensure accurate representation of participant experiences.

We used the eight criteria (worthy topic, rich rigor, sincerity, credibility, resonance, significant contribution, ethical, meaningful coherence) established by Tracy to ensure high quality in our analysis process [33]. Our research topic was considered worthy given the prevalence of perinatal mental health disorders in SA. We ensured rigor in our data collection by co-creating the semi-structured guide with our SA-based team and iteratively refining the guide to meet the study aims and to provide rich data. In our analysis, we achieved rigor by following the theories of thematic analysis and creating a thorough codebook, with feedback from our SA-based collaborators across multiple iterations, to accurately capture the data. Sincerity was continuosly assessed and ensured by recognizing our team’s biases and the limitations of the US-based team with respect to cultural understanding throughout the analysis process, as well as by transparently describing our methods. Credibility was reached through detailed descriptions of the findings and triangulating conclusions with multiple study team coders. Resonance, or the ability for the research to influence the audience, was achieved by creating a captivating narrative through carefully selected quotes that represent participants’ lived experiences. This study contributed significantly to our current knowledge of how pregnant women anticipate that their depression and/or PTSD symptoms will impact their postpartum caregiving. We ensured alignment with ethical procedures through ethical review and approval, relationally with both our researchers and participants, situationally and culturally (as guided by our team in SA), and in study closeout through our data dissemination efforts. We finally met the eighth criterion, meaningful coherence, by interpreting our results in the context of the existing literature.

In alignment with Patton’s approach, we based our sample size for the qualitative analysis on the richness of data captured and the analytic abilities of the research team, rather than relying on the principles of thematic saturation [34].

## Results

### Characteristics of sample

Overall, 23 participants completed 30 interviews (17 completed an interview during pregnancy, and 13 completed a postpartum interview, with seven participants completing interviews at both timepoints) between May 2022 and May 2023. On average, participants were 25.1 years old (SD=4.0), had a mean gestational age of 28.5 weeks (SD = 9.9), and had 2.1 (SD=1.0) lifetime pregnancies at the time of their baseline survey (see Table 1 for full details). Most participants identified as Black South African (n=20, 83.3%), and 70.8% of the sample (n=17) earned less than 3,000 ZAR (~$163) per month. With respect to likely mental health diagnoses, 12 met criteria for probable depression, two for probable PTSD, and nine for both.

In addition to topics related to PrEP use that are not discussed here, the qualitative interviews explored perspectives on the likely impact of partcipants’ mental health symptoms on their baby’s wellbeing, their ability to bond with their baby, and their ability to meet their baby’s needs (i.e., feeding and taking their baby to medical appointments). Three main themes that characterized these relationships emerged from the data: (1) a strong perceived connection between maternal mental health and baby’s wellbeing; (2) perceived strains on bonding with the baby; and (3) negative impact of mental health on likelihood of completing parenting tasks completing parenting tasks.

#### Theme 1: A Strong Perceived Connection Between Mental Health & Baby’s Wellbeing

Most participants endorsed a strong connection between their mental health and their baby’s wellbeing, both *in utero* and post-delivery. Two subthemes that described how participants understood their mental health to impact their baby’s health were identified: (1a) fear of mental health symptoms impairing their baby’s health through birth complications and via breastmilk, and (1b) avoidance of sadness (either cognitively or emotionally) as a strategy to protect their baby.

##### (1a) Fear of mental health symptoms impairing baby’s health through birth complications and via breastmilk.

Participants perceived a connection between their mental health symptoms and the possibility of pregnancy complications, including risk of pregnancy loss or miscarriage. For example, one participant stated, “People say that if you always cry it might affect the child, and you might get miscarriage, and that’s why I don’t think much of the [bad things] that happen…That’s why I don’t think much because I’m concerned about the baby that’s inside me” (age 21, pregnant). Another participant noted, “When I think a lot; I become very angry and hurt. I heard that in most cases when someone is going through stressful phase; miscarriage occurs” (age 22, pregnant). These concerns about symptoms leading to a potential miscarriage demonstrate a clear perceived link between maternal stress, depression symptoms, and severe consequences for the baby.

Further, participants also identified a perceived connection between their mental health symptoms and resulting complications for their baby postpartum. One participant shared, “Oh no [my stress] affects me just a little bit until I realize that the baby must not see me. It’s said that the baby can sense when you’re not alright” (age 31, postpartum). This comment suggests the social pressure of ensuring that the baby is not aware of or attuned to their mother’s negative emotions and/or thoughts. Another participant expressed a similar sentiment, and revealed that her mother told her that stress can meaningfully affect the health of the baby, “My mother would tell me that I should decrease my stress levels because the more I get stressed, this is going to affect the baby, she said I should stop having negative thoughts” (age 23, postpartum). Many participants were made aware of the connection between their mental health symptoms and their baby’s wellbeing from relatives or others in their community, which strengthened beliefs that their negative emotions or feelings may negatively impact their baby.

Participants were also told that their sadness or stress could transfer to their baby when breastfeeding postpartum. One postpartum participant explained, “When it’s time to breastfeed the baby, it is normally said that if you’re upset you must not breastfeed the baby” (age 31, postpartum). Taken together, particpants recognized the potential for their mental health symptoms to impact their baby’s health both *in utero* and while breastfeeding.

##### (1b) Avoidance of sadness (either cognitively or emotionally) was seen as a strategy to protect their baby

Participants discussed cognitively or emotionally avoiding their mental health symptoms to protect their baby during pregnancy. One participant mentioned actively trying to reduce her overthinking: “Things do happen, but we must not think too much of them, and they would say I must not think too much because it will get me sick and increase my blood pressure” (age 25, pregnant). This participant tried to avoid negative cognitions because she was told by others that the resulting physical effects will cause harm to both her and the baby. In a similar way, another participant stated, “My baby’s happiness comes first, so if I worry myself, that is also going to affect my baby and the nurses would notice this. So, I will try not to think about those things, let’s say I try to pretend…I will try to be present, just for my baby’s sake” (age 20, pregnant). This participant described the process of compartmentalizing her stress or “pretending” she was not experiencing anxiety toto protect her baby’s health and because she believed that the clinic nurses would be able to notice in her baby if she had been stressing..

Participants also believed they must continue to avoid or suppress their mental health symptoms while breastfeeding. One participant with likely posttraumtic stress clarified that when she has stress-related thoughts, she tries to ignore her negative cognitions so her baby is not impacted, “I don’t think a lot now like how I used to…Reason being, it’s said when you are breastfeeding the baby can sense when you’re stressed…So, whenever I have that feeling, I ignore it” (age 27, postpartum). This participant believed she must put aside her negative thoughts and associated distress to prioritize feeding her baby. Collectively, participants were highly aware of potential negative impacts of their mental health symptoms on their baby’s wellbeing, and they highlighted the importance of ensuring that their babies were protected, even if it was challenging to do so.

#### Theme 2: Perceived Strains on Bonding with the Baby

The second main theme that emerged was strains on bonding between mother and baby, with respect to the impact of mental health symptoms on the emotional aspects of parenting. Participants discussed two specific ways in which mental health contributed to strains on emotional bonding, both during pregnancy and postpartum: (2a) via increased anger and/or (2b) through a lack of emotional connection. A third subtheme emerged that highlights one pathway that may protect against strains on emotional bonding with the baby: (2c) a strong desire to parent in a way that breaks the cycle of their own negative experiences with their parents and/or other family members.

##### (2a) Increased anger and irritability

Some particpants experienced increased anger due to life stressors and current mental health symptoms, which either led to predictions that bonding might be negatively affected or negatively impacted bonding postpartum. One participant described the anticipated effects of anger on her feelings towards and interactions with her baby:

I don’t think I will have a relationship with my baby because I will look at him/her with angry eyes. I think I will not cope to do anything in his/her presence; I feel I will just burst in tears (age 22, pregnant).

Not only does this participant expect that her anger will impair their bond, she also predicts that she will not be able to cope or find strength in her baby’s presence, suggesting that the baby may continue to serve as a reminder of negative emotions or previous experiences.

Similarly, another participant felt that the stress associated with her life circumstances impacted her self-perception as a parent. She explained, “When I shout at [my other daughter] I feel like beating her up, but I am avoiding that. I don’t think I am a bad parent, but I feel like I am dealing with a lot” (age 24, pregnant). This finds herself projecting her anger onto her children, which she does not want to do. She copes by rationalizing that she is a good mother who is trying to handle a number of stressors. She expressed that this process may impact her ability to form a strong, longstanding emotional connection with her baby and her other children.

##### (2b) Lack of emotional connection

In addition to the negative impacts of anger on bonding, some participants felt that their anhedonia would result in no connection, or a very limited connection, with their babies. Among pregnant participants, primarily, current stress or sadness fueled the sense of emotional distance. One participant shared, “I think I won’t have a bond with my baby” (age 30, pregnant), and another stated, “I won’t have a relationship with this child, I don’t think I will” (age 21, pregnant). Due to feelings of sadness, other forms of negative affect, or general negative thoughts, participants anticipated forming no bond with their baby once they were born. This lack of bonding or emotional withdrawal could then exacerbate participants’ symptoms of depression, knowing they are not able to provide the care for their baby that they would like to. One participant explained, “I am not going to feel alright because the baby has to be taken care of, and yet I would be feeling like this feeling down and not wanting to talk” (age 28, pregnant). This participant is articulating that withdrawal, a primary symptom of depression, will ultimately prevent her from properly taking care of and communicating with her baby, which would, in turn, exacerbate her sadness.

##### (2c) A desire to parent in a way that breaks generational cycles

Importantly, although many participants assumed that they might either have an anger-driven relationship with their baby or no emotional connection at all, some participants were more likely to highlight that they would actively seek to build stronger connections with their babies compared to the connection they had with key adult figures when they were growing up. For example, several participants expressed a desire to break the negative generational cycle of poor parenting or abuse. This manifested through expressions of resilience and optimism about raising their babies in the context of very difficult personal circumstances and poor modeling from their own families. One participant described a discussion that she had with her own mother, which helped her clarify how she will make key parenting decisions:

[My mother] said something that made me realize that I don’t want to be like her. She told me since she had me when she was in high school, she said ‘I didn’t even want to fall pregnant. I didn’t even want you. If I had a choice, I would have terminated you, but because of my mother, meaning my grandmother I couldn’t. So, I hate you, I don’t like you at all’. So even if I am sad, I don’t want to end the pregnancy because I feel like my child is my world. There’s no one who loves me, but I feel like this child is going to love me (age 24, pregnant).

This participant’s lack of bond with her mother, paired with the sense that she did not receive the love that she wanted and deserved, profoundly influenced how she views her relationship with her future baby. This excerpt also displays the ways in which vicarious learning from previous generations influences parenting styles; she believes that her sadness will not interfere with her own loving relationship with her baby.

Other participants also endorsed a sense of hope and a desire to be strong for their babies, primarily because they wanted to be better parents than their parents. One participant articulated, “I am going to take care and show love to my baby because I don’t want my baby to have the anger I had, because I never received love from my parents” (age 25, pregnant). This is another example of how intergenerational negative experiences can impact the relationship between the mother and new baby; the participant decided to care for and love her baby, despite her anger, so that she may provide a more supportive upbringing than she experienced. Similarly, another participant shared, “I must be strong for my baby, I don’t know how to explain this, that’s where I get hope that I should leave everything in the past and just take care of my child” (age 25, postpartum). She draws strength from remembering her own upbringing and actively choosing to be a supportive mother for her own baby. Overall, participants expressed that their mental health symptoms may strain their relationship with their babies in different ways. However, some found resilience and hope for emotional connection out of a commitment to avoid reinforcing generational patterns of parenting that left them feeling unloved.

#### Theme 3: Negative Impact of Mental Health on Likelihood of Completing Parenting Tasks

In the context of mental health symptoms, participants anticipated challenges completing practical parenting tasks (e.g., feeding the baby, taking the baby to medical appointments) through the following pathways: (3a) reduced ability to or decreased likelihood of completing the tasks, and (3b) an increased sense of obligation to care for and prioritize the baby above all else, including intentional efforts to put the practical parenting tasks first despite their psychological distress; and (3c) finding sources of support when overcoming mental health symptoms to caregive.

##### (3a) Reduced ability to or decreased likelihood of completing the tasks

Most participants expected that their ability to complete practical parenting tasks would be disrupted, given their current mental health challenges. Some participants indicated that their ability to feed their baby would be impaired. For example, one participant expressed that her milk production was previously inhibited by her stress levels: “It affects me because I can’t eat when stressed, I don’t produce enough milk for the baby, and I don’t have the patience” (age 30, pregnant). This comment highlights the strength of the association between poor mental health and physiological challenges or complications, much like the connection linking symptoms to likely miscarriage described previously. Other participants shared that their sadness would lead them to forget to feed their babies: “I am not going to feed my baby when I am feeling sad; I might as well forget to feed her” (age 30, pregnant). Not only will mental health symptoms inhibit milk production, but symptoms will also reduce attentiveness to their baby’s needs (i.e., providing nourishment).

Several participants also shared that their mental health symptoms would inhibit their ability to leave the house and attend appointments at the antenatal clinic. As a result of previous trauma, one participant worried that another distressing event would happen when she left the house to go to the clinic: “[My sadness] does affect me because now I don’t like being around other people. When I’m around people I would fear that something bad is going to happen because of thinking too much” (age 28, pregnant). This participant expected that their symptoms of withdrawal and avoidance to inhibit their ability to attend clinic. Similarly, another participant expressed,

I’m always uncomfortable when I come to the clinic, I’m scared that they might break-in my house, when they did that it was during the day, so I am always worried… I must make sure I rush back home. That disturbs me a lot, even when I’m at the clinic I must think about what might happen (age 25, postpartum).

This participant expressed increased stress and fear upon leaving her house in the aftermath of a traumatic house break-in, describing the ways in which her posttraumatic stress symptoms may impair her ability to attend to her baby’s medical needs.

##### (3b) An increased sense of obligation to care for and prioritize the baby

Conversely, some participants felt a sense of obligation or sacrifice to make sure that their baby’s needs will be met. One participant shared that her mental health symptoms would negatively affect the baby if she does not prioritize the baby above all else, at all times, despite her mental health challenges: “It is going to affect the baby because I have to take care of the baby at all times and if I don’t, this is going to affect the baby negatively” (age 28, pregnant). For the wellbeing of her baby, she believed she must overcome her mental health symptoms or put them aside to focus on the baby. When asked about how her mental health symptoms may impact feeding her baby, one participant described, “I will bottle-feed my unborn baby; I breastfeed [my first child] even when I am sad” (age 26, pregnant), indicating that her parental obligation would supercede any negative impacts of her symptoms. Another participant stressed this strong sense of obligation, “There is nothing I can do. I have to think about my baby, I have to feed the baby even if I have stress, I have to take care of my baby” (age 30, pregnant). Many participants used phrases like “I love my baby” and “for the sake of my baby” to describe their responsibility to bring their baby to the clinic. For example: “I will attend the clinic appointment because I love my baby, I have to sacrifice even if I’m feeling sad” (age 30, pregnant). The love and sense of duty as a new mother acts as a driving force to overcome symptoms of depression or stress that may otherwise impair their ability to complete everyday tasks.

##### (3c) Finding sources of support when overcoming mental health symptoms to caregive

In some cases, the sense of personal obligation or sacrifice required to parent led to the discovery of unanticipated sources of social support in other mothers. One participant discussed the importance of taking her baby to the clinic and going against the instincts to withdraw or avoid leaving the house:

I decided to come to the clinic so that my baby can be in good health…and I have gained a lot from talking to others [at the clinic], and sometimes you might think you are the only one with the problem but that is not true, some people have worse stories than yours. Then you must just take your treatment and move on with your life (age 31, pregnant).

While attending the clinic, she experienced the benefits of interacting with others in the queue and building community. Her interactions while bringing her baby to the clinic improved her own mental status. Other participants indicated that their stress was reduced at the clinic due to the support of nurses and staff. One participant went on to say, “It is much better when I am here [at the clinic]. I adhere properly [to appointments]. I enjoyed attending, because it is much better when I am here than being at home” (age 31, postpartum); “I feel like the clinic is a good place for my baby…it’s stressless for me and stress more for the nurses because they are the ones who must find out what is happening with my baby” (age 31, pregnant). This participant explains that bringing her baby to the clinic would put her at ease, rather than cause her more stress. She felt supported by the nurses and clinic staff to help her keep her baby healthy.

## Discussion

This study explored perceptions of the impact of depression and PTSD symptoms on caregiving and bonding with their infants among pregnant and postpartum women in SA. Three main themes emerged: a strong perceived connection between maternal mental health and baby’s wellbeing; (2) perceived strains on bonding with the baby; and (3) negative impact of mental health on likelihood of completing parenting tasks. These results provide additional insight on the ways in which women expect their mental health symptoms to compromise their caregiving abilities, and also how a sense of resilience can contribute to overcoming psychological barriers in order to provide for the baby.

First, participants expressed their belief in a strong connection between their own mental health and their baby’s health. This is supported by the literature; there is growing evidence of the link connecting maternal stress and depression to poor pregnancy outcomes. For example, there is a strong association between maternal stress and the development of hypertensive disorders during pregnancy, including pre-eclampsia and eclampsia [35]. There is also evidence that maternal psychological stress is associated with an increased risk of miscarriage [36]. It is likely that efforts to inform pregnant women about the risks of maternal anxiety and depression have contributed to women’s perspectives on how their mental health impacts their baby. However, there is a lack of published literature, particularly qualitative perspectives, from women on the impact their mental health has on their baby’s wellbeing. For this reason, our study meaningfully offers perspectives on how women perceive their poor mental health to negatively impact the wellbeing of their baby *in utero* and while breastfeeding, and how through cognitively avoiding their depression or stress, they hope to protect their baby.

Participants also discussed expectations and experiences of strains on bonding between mother and baby, with respect to the impact of mental health symptoms on the emotional aspects of parenting. The maternal-infant emotional relationship plays a large role in healthy development for a child. Bigelow and colleagues have discussed how maternal sensitivity (i.e., appropriately responding to cues from the child) impacts secure attachment, both of which are associated with positive developmental outcomes [37]. Our findings align with research on common maternal mental health disorders and the maternal-infant relationship. For example, perinatal mental health disorders can impair a mother’s ability to bond with their infant, including acting negatively towards the child [38], and negative cognitions associated with depression (e.g., self-focus, psychological distancing) can contribute to challenges understanding the baby’s emotions and impair caregiver warmth [39]. Worry or rumination can also impair the maternal-child relationship [40, 41], and in more severe cases, anhedonia has been linked with emotional neglect of a child [42]. It is also now known that most mental health disorders have their origins in childhood and adolescence, which reinforces the importance of maintaining a healthy environment for child development [43]. Interestingly, in the present study, some participants also discussed a desire to break the negative cycle of poor parenting that they experienced. These results suggest how an increased sense of resilience can prevent mental health symptoms from impairing the bond between parent and infant.

In this sample, the negative impact of mental health symptoms on completing parenting tasks aligned with previous papers on maternal mental health and sensitivity. Since mental health symptoms have been found to be associated with decreased maternal sensitivity, this may be one mechanism driving the relationship between mental health symptoms and unmet infant needs [39]. Mental health symptoms have also been shown to impair sustainable feeding practices, such that postpartum depression is one of the main factors influencing breastfeeding cessation [44]. Participants in this study openly discussed their stress or sadness as an expected barrier to feeding, though a separate SA-based study found no significant associations between poor breastfeeding outcomes and mental health [45]. Breastfeeding, which has a complex relationship with maternal mental health, has also been shown to have a protective effect against mental health symptoms, primarily through the release of oxytocin [46–48]. Exclusive breastfeeding is recommended for the first six months for persons with and without HIV by the WHO, though the traditional “breast is best” narrative is oversimplified, as recommendations should be tailored to the physical and mental health needs of the mother [49–51]. For example, women who face physical challenges or pain when trying to exclusively breastfeed may be more likely to experience depression [52]. Supporting mothers facing common mental health disorders through breastfeeding may be an important way to improve their symptoms and improve their relationship with their baby.

Some participants discussed how they coped with their mental health symptoms to fulfill a strong obligation to care for their baby. They found strength, or expected to find strength, in providing for their new baby through a strong sense of responsibility as a mother. This was found to be true when discussing breastfeeding, providing general care, and attending medical appointments. In SA, it is a large commitment to attend appointments at the clinic due to far walking distances, risk of harm when traveling through neighborhoods and using transit that is not safe, taking time away from work or family commitments, and waiting in long queues to be seen. One study in Ethiopia found that maternal depression, long walking distances, and late initial engagement in antenatal care predicted inadequate service utilization among antenatal patients [12]. That said, many participants in this study discussed overcoming or coping with their mental health challenges in order to come to the clinic. They were strongly motivated to provide for their babies, putting their baby’s needs first in almost all circumstances.

When overcoming their mental health symptoms, some participants found new support systems, particularly at the antenatal care clinic. Participants discussed connecting with other patients at the clinic and how clinic staff relieved their stress over health concerns for their baby. A qualitative study conducted in Johannesburg, SA found when participants received informational support in health settings, they were more likely to think positively of their pregnancy [53]. A strong support network is critical for the wellbeing of pregnant and postpartum persons, and those who have this support are more likely to embrace their pregnancy [53]. Outside of family, partners, neighbors, or peers, attending the clinic seemed to provide another level of support to participants as they prepared to care for their baby.

### Limitations

It is important to note a few limitations of the current study. First, participants were recruited from an antenatal clinic, meaning that the sample only included pregnant and postpartum women who were already engaged in care. These women were therefore already exhibiting resilience with respect to their mental health symptoms. This study also shares limitations that are common among secondary analyses of qualitative data; the interview guide was already written and the interview data were collected when the analysis began; therefore, the guide was not developed to specifically address the main question of these analyses, and we were unable to probe around topics like the perceived impact of mental health on bonding. We were also unable to interview all participants at both time points due to retention challenges postpartum. The parent study was designed to explore psychological barriers to PrEP uptake during pregnancy and after delivery, but only seven of the 23 participants completed two interviews (one during preganancy and one postpartum). Therefore, we could not formally assess differences in perceived impacts of mental health on caregiving and bonding between pregnancy and postpartum, though some participants who were interviewed only postpartum did reflect on differences. Finally, some meaning and cultural nuances may have been lost in translation, as most interviews were conducted in isiXhosa and then analyzed in English after transcription and translation. We worked closely with our isiXhosa-speaking team-members to reduce the likelihood of losing key information and cultural context during the analysis and interpretation phases, but it is possible that some cultural nuances may have been missed or misinterpreted.

### Implications

Given the relevance of maternal mental health for both the mother and the baby’s wellbeing, it is critical to provide pregnant and postpartum persons with mental, physical, and social support. In SA, this support will likely contribute meaningfully to HIV prevention efforts during pregnancy and breastfeeding [54]. A parent’s ability to attend clinic appointments, nourish their baby, and engage in HIV prevention behaviors, such as taking pre-exposure prophylasis consistently during the peripartum period, may well reduce the risk of HIV acquisition and transmission as well as support the wellbeing of the infant.

Leveraging these insights to support mental health programming and intervention development for pregnant people who are at risk for acquiring HIV will enable the execution of key policies and strategic initatives that have been championed by the SA government. For example, the South African National Mental Health Policy Framework and Strategic Plan 2023–2030 states that action is required to develop mental health promotion/mental illness prevention programs, particularly at key developmental life stages including pregnancy, early childhood, adolescence, and old age [55]. Recently, the Practical Approach to Care Kit (PACK), a tool kit from the University of Cape Town, was developed to support clinical decision making and standardize care, including mental health care, in low- and middle-income primary care settings [56]. However, implementation challenges remain. Despite the strong need for interventions to improve peripartum mental health and associated HIV-outcomes [57], there are still many barriers to routine mental health screening, psychoeducation, and efficient treatment referral pathways in South African antenatal clinics [58].

## Conclusion

Pregnant and postpartum persons perceived that symptoms of depression and PTSD may impair the emotional and practical aspects of parenting. While most expected their mental health symptoms to compromise their ability to caregive, some expressed a sense of resilience to overcome and/or de-prioritize their mental health symptoms for the sake of their babies and in defiance of established generational patterns. Our findings support the need for improved access to mental health screening and treatment in the antenatal care setting, and may be well placed to inform future mental health interventions for peripartum persons in SA, especially given the potential for inter-generational effects and for negative, HIV-related public health impacts.

## Supplementary Material

Supplement 1

## Figures and Tables

**Table 1.** Sociodemographic and select characteristics of sample at baseline survey visit (N = 23)

	Mean	SD
Age	25.1	4.0

Gestational age (wks) at baseline visit	28.5	9.9

Gestational age (wks) at time presenting for antenatal care	17.6	8.7

Total pregnancies, including current	2.1	1.0

Previous live births	1.7	1.0

Mental health symptom severity		
Depression (SADS)	24.2	8.0
Depression (EPDS)	11.1	4.7
PTSD (PCL-5)	28.0	13.6
	N	%

Race		
Black South African	20.0	87.0
Black non-South African	3.0	13.0

Education		
None	1.0	4.4
Through Grade 10/Std 8	4.0	17.4
Through Grade 12/Std 10	15.0	65.2
Vocational	2.0	8.7
Tertiary, university, Technikon degree	1.0	4.4

Monthly income in ZAR ($)		
0 (0 USD)	5.0	21.7
Less than 3000 (162 USD)	11.0	47.8
3001–6000 (162–324 USD)	6.0	26.1
6001–9000 (324–485 USD)	0.0	0.0
9001–12000 (485–647 USD)	1.0	4.4

Source of income		
Employment	8.0	34.8
Government grant	9.0	39.1
Money from partner(s)	4.0	17.4
Money from family	2.0	8.7
Other	2.0	8.7
None	5.0	21.7

Qualified for interview by		
Depression	13.0	56.5
Posttraumatic stress	3.0	13.0
Depression and posttraumatic stress	7.0	30.0

## Data Availability

We are prepared to send relevant documentation or de-identified data in order to verify the validity of the results presented.

## References

[1] Mokwena K, Masike I. The Need for Universal Screening for Postnatal Depression in South Africa: Confirmation from a Sub-District in Pretoria, South Africa. International Journal of Environmental Research and Public Health [Internet]. 2020 Oct [cited 2024 Feb 15];17(19). Available from: https://www.ncbi.nlm.nih.gov/pmc/articles/PMC7579387/10.3390/ijerph17196980 PMC7579387 32987674

[2] Roddy Mitchell A, Gordon H, Lindquist A, Walker SP, Homer CSE, Middleton A, Prevalence of Perinatal Depression in Low- and Middle-Income Countries. JAMA Psychiatry. 2023 May;80(5):425–31. 36884232 10.1001/jamapsychiatry.2023.0069 PMC9996459

[3] Redinger S, Norris SA, Pearson RM, Richter L, Rochat T. First trimester antenatal depression and anxiety: prevalence and associated factors in an urban population in Soweto, South Africa. J Dev Orig Health Dis. 2018 Feb;9(1):30–40. 28877770 10.1017/S204017441700071X

[4] Hartley M, Tomlinson M, Greco E, Comulada WS, Stewart J, le Roux I, Depressed mood in pregnancy: prevalence and correlates in two Cape Town peri-urban settlements. Reprod Health. 2011 May 2;8:9. 21535876 10.1186/1742-4755-8-9 PMC3113332

[5] van Heyningen T, Honikman S, Myer L, Onah MN, Field S, Tomlinson M. Prevalence and predictors of anxiety disorders amongst low-income pregnant women in urban South Africa: a cross-sectional study. Arch Womens Ment Health. 2017 Dec;20(6):765–75. 28852868 10.1007/s00737-017-0768-z PMC6086488

[6] Brown S, MacNaughton G, Sprague C. A Right-to-Health Lens on Perinatal Mental Health Care in South Africa. Health Hum Rights. 2020 Dec;22(2):125–38. 33390702 PMC7762903

[7] Tomlinson M, O’Connor MJ, le Roux IM, Stewart J, Mbewu N, Harwood J, Multiple risk factors during pregnancy in South Africa: the need for a horizontal approach to perinatal care. Prev Sci. 2014 Jun;15(3):277–82. 23475562 10.1007/s11121-013-0376-8 PMC3718865

[8] Kaminer D, Grimsrud A, Myer L, Stein DJ, Williams DR. Risk for post-traumatic stress disorder associated with different forms of interpersonal violence in South Africa. Social Science & Medicine. 2008 Nov;67(10):1589–95. 18774211 10.1016/j.socscimed.2008.07.023 PMC2610682

[9] Peltzer K, Pengpid S, McFarlane J, Banyini M. Mental health consequences of intimate partner violence in Vhembe district, South Africa. General Hospital Psychiatry. 2013 Sep;35(5):545–50. 23643034 10.1016/j.genhosppsych.2013.04.001

[10] Bernard C, Dabis F, De Rekeneire N. Prevalence and factors associated with depression in people living with HIV in sub-Saharan Africa: A systematic review and meta-analysis. Seedat S, editor. PLoS ONE. 2017 Aug 4;12(8):e0181960. 28783739 10.1371/journal.pone.0181960 PMC5544236

[11] National Institute of Mental Health (NIMH). Perinatal Depression [Internet]. 2023 [cited 2024 Aug 5]. Available from: https://www.nimh.nih.gov/health/publications/perinatal-depression

[12] Beyene GM, Azale T, Gelaye KA, Ayele TA. Effect of antenatal depression on ANC service utilization in northwest Ethiopia. Sci Rep. 2023 Sep 2;13:14443. 37660079 10.1038/s41598-023-37382-9 PMC10475009

[13] Joyce K, Diffenbacher G, Greene J, Sorokin Y. Internal and External Barriers to Obtaining Prenatal Care. Social Work in Health Care. 1984 Jan 20;9(2):89–96. 10.1300/j010v09n02_09 6670049

[14] Christie H, Hamilton-Giachritsis C, Alves-Costa F, Tomlinson M, Halligan SL. The impact of parental posttraumatic stress disorder on parenting: a systematic review. Eur J Psychotraumatol. 10(1):1550345. 10.1080/20008198.2018.1550345 PMC6338266 30693071

[15] Schwab W, Marth C, Bergant AM. Post-traumatic Stress Disorder Post Partum. Geburtshilfe Frauenheilkd. 2012 Jan;72(1):56–63. 25253905 10.1055/s-0031-1280408 PMC4168363

[16] Koen N, Brittain K, Donald KA, Barnett W, Koopowitz S, Maré K, Maternal posttraumatic stress disorder and infant developmental outcomes in a South African birth cohort study. Psychological Trauma: Theory, Research, Practice, and Policy. 2017 May;9(3):292–300. 28459271 10.1037/tra0000234 PMC5461402

[17] Dadi AF, Akalu TY, Wolde HF, Baraki AG. Effect of perinatal depression on birth and infant health outcomes: a systematic review and meta-analysis of observational studies from Africa. Arch Public Health. 2022 Dec;80(1):34. 35057865 10.1186/s13690-022-00792-8 PMC8772173

[18] Kendig S, Keats JP, Hoffman MC, Kay LB, Miller ES, Moore Simas TA, Consensus Bundle on Maternal Mental Health: Perinatal Depression and Anxiety. Obstetrics & Gynecology. 2017 Mar;129(3):422–30. 28178041 10.1097/AOG.0000000000001902 PMC5957550

[19] Slomian J, Honvo G, Emonts P, Reginster JY, Bruyère O. Consequences of maternal postpartum depression: A systematic review of maternal and infant outcomes. Womens Health (Lond). 2019;15:1745506519844044. 31035856 10.1177/1745506519844044 PMC6492376

[20] Cooper P, Tomlinson M, Swartz L, Woolgar M, Murray L, Molteno C. Post partum depression and the mother-infant relationship in South Africa peri-urban settlement. The British journal of psychiatry : the journal of mental science. 2000 Jan 1;175:554–8. 10.1192/bjp.175.6.554 10789353

[21] Murakami K, Ishikuro M, Obara T, Noda A, Ueno F, Onuma T, Maternal postnatal bonding disorder and emotional/behavioral problems in preschool children: The Tohoku Medical Megabank Project Birth and Three-Generation Cohort Study. Journal of Affective Disorders. 2023 Mar;325:582–7. 36642309 10.1016/j.jad.2023.01.044

[22] Nurius PS, Green S, Logan-Greene P, Borja S. Life course pathways of adverse childhood experiences toward adult psychological well-being: A stress process analysis. Child Abuse & Neglect. 2015 Jul;45:143–53. 25846195 10.1016/j.chiabu.2015.03.008 PMC4470711

[23] Bell J, Norris S, Shand A, Lain S, Hall B, Healthy mothers and babies - a lifecourse approach: an Evidence Check rapid review brokered by the Sax Institute for the NSW Ministry of Health [Internet]. 2018 [cited 2024 May 31]. Available from: https://www.saxinstitute.org.au/wp-content/uploads/2019_Healthy-mothers-and-babies-a-life-course-approach.pdf

[24] Andersen LS, Joska JA, Magidson JF, O’Cleirigh C, Lee JS, Kagee A, Detecting Depression in People Living with HIV in South Africa: The Factor Structure and Convergent Validity of the South African Depression Scale (SADS). AIDS Behav. 2020 Aug;24(8):2282–9. 31965430 10.1007/s10461-020-02787-4 PMC8021389

[25] Cox JL, Holden JM, Sagovsky R. Detection of postnatal depression. Development of the 10-item Edinburgh Postnatal Depression Scale. Br J Psychiatry. 1987 Jun;150:782–6. 3651732 10.1192/bjp.150.6.782

[26] Rodriguez V, Mandell L, Jones D. Factor Structure and Differential Item Functioning of the Edinburgh Postnatal Depression Scale: A Comparison of Zulu and English Versions Among Ante- and Postnatal Women Living with HIV in South Africa. Maternal and Child Health Journal. 2022 Jul 1;26. 10.1007/s10995-022-03418-1 PMC9372781 35303221

[27] Redinger S, Pearson RM, Houle B, Norris SA, Rochat TJ. Antenatal depression and anxiety across pregnancy in urban South Africa. J Affect Disord. 2020 Dec 1;277:296–305. 32858310 10.1016/j.jad.2020.08.010

[28] Blevins CA, Weathers FW, Davis MT, Witte TK, Domino JL. The Posttraumatic Stress Disorder Checklist for DSM‐5 (PCL‐5): Development and Initial Psychometric Evaluation. Journal of Traumatic Stress. 2015 Dec;28(6):489–98. 26606250 10.1002/jts.22059

[29] Huberman A, Miles M. The Qualitative Researcher’s Companion [Internet]. 2455 Teller Road, Thousand Oaks California 91320 United States of America: SAGE Publications, Inc.; 2002 [cited 2024 May 31]. Available from: https://methods.sagepub.com/book/the-qualitative-researchers-companion

[30] Braun V, Clarke V. Thematic analysis. In: Cooper H, Camic PM, Long DL, Panter AT, Rindskopf D, Sher KJ, editors. APA handbook of research methods in psychology, Vol 2: Research designs: Quantitative, qualitative, neuropsychological, and biological [Internet]. Washington: American Psychological Association; 2012 [cited 2023 Aug 7]. p. 57–71. Available from: http://content.apa.org/books/13620-004

[31] Glaser BG, Strauss AL. The discovery of grounded theory: strategies for qualitative research. London New York: Routledge; 2017. 271 p.

[32] Dedoose [Internet]. Los Angeles, CA: SocioCultural Research Consultants, LLC; 2021. (cloud application for managing, analyzing, and presenting qualitative and mixed method research data). Available from: www.dedoose.com

[33] Tracy SJ. Qualitative Quality: Eight “Big-Tent” Criteria for Excellent Qualitative Research. Qualitative Inquiry. 2010 Dec 1;16(10):837–51.

[34] Patton MQ. Qualitative Research & Evaluation Methods: Integrating Theory and Practice [Internet]. Fourth edition. Saint Paul, MN: Sage Publications; 2014 [cited 2023 Feb 9]. Available from: https://us.sagepub.com/en-us/nam/qualitative-research-evaluation-methods/book232962

[35] Raina J, El-Messidi A, Badeghiesh A, Tulandi T, Nguyen TV, Suarthana E. Pregnancy hypertension and its association with maternal anxiety and mood disorders: A population-based study of 9 million pregnancies. Journal of Affective Disorders. 2021 Feb;281:533–8. 33388464 10.1016/j.jad.2020.10.058

[36] Qu F, Wu Y, Zhu YH, Barry J, Ding T, Baio G, The association between psychological stress and miscarriage: A systematic review and meta-analysis. Sci Rep. 2017 May 11;7(1):1731. 28496110 10.1038/s41598-017-01792-3 PMC5431920

[37] Bigelow AE, MacLean K, Proctor J, Myatt T, Gillis R, Power M. Maternal sensitivity throughout infancy: Continuity and relation to attachment security. Infant Behavior and Development. 2010 Feb;33(1):50–60. 20004022 10.1016/j.infbeh.2009.10.009

[38] World Health Organization. Maternal mental health and child health and development in low and middle income countries : report of the meeting, Geneva, Switzerland, 30 January - 1 February, 2008. 2008; Available from: https://iris.who.int/handle/10665/43975

[39] Humphreys K, King L, Choi P, Gotlib I. Maternal depressive symptoms, self-focus, and caregiving behavior. Journal of Affective Disorders. 2018 Jun 1;238. 10.1016/j.jad.2018.05.072 PMC6604802 29929156

[40] Stein A, Craske MG, Lehtonen A, Harvey A, Savage-McGlynn E, Davies B, Maternal cognitions and mother–infant interaction in postnatal depression and generalized anxiety disorder. Journal of Abnormal Psychology. 2012 Nov;121(4):795–809. 22288906 10.1037/a0026847 PMC3506203

[41] Tester‐Jones M, Karl A, Watkins E, O’Mahen H. Rumination in dysphoric mothers negatively affects mother–infant interactions. Child Psychology Psychiatry. 2017 Jan;58(1):38–45. 10.1111/jcpp.12633 27616434

[42] van Veen T, Wardenaar KJ, Carlier IVE, Spinhoven P, Penninx BWJH, Zitman FG. Are childhood and adult life adversities differentially associated with specific symptom dimensions of depression and anxiety? Testing the tripartite model. J Affect Disord. 2013 Apr 5;146(2):238–45. 23084183 10.1016/j.jad.2012.09.011

[43] Kessler RC, Amminger GP, Aguilar-Gaxiola S, Alonso J, Lee S, Ustün TB. Age of onset of mental disorders: a review of recent literature. Curr Opin Psychiatry. 2007 Jul;20(4):359–64. 17551351 10.1097/YCO.0b013e32816ebc8c PMC1925038

[44] Stelson EA, Kulkacek L, Frasso R, Hall M, Guevara JP. Perspectives on Breastfeeding from Mothers with Postpartum Depression Symptoms: A Qualitative Assessment of Antecedents, Barriers, Facilitators, and Intervention Suggestions. Breastfeeding Medicine. 2021 Oct 1;16(10):790–8. 34010030 10.1089/bfm.2020.0251 PMC8817733

[45] Thomas E, Kuo C, Cohen S, Hoare J, Koen N, Barnett W, Mental health predictors of breastfeeding initiation and continuation among HIV infected and uninfected women in a South African birth cohort study. Preventive Medicine. 2017 Sep;102:100–11. 28694059 10.1016/j.ypmed.2017.07.004 PMC5802398

[46] Anoop S, Saravanan B, Joseph A, Cherian A, Jacob K. Maternal depression and low maternal intelligence as risk factors for malnutrition in children: a community based case-control study from South India. Arch Dis Child. 2004 Apr;89(4):325–9. 15033840 10.1136/adc.2002.009738 PMC1719856

[47] Rahman A, Harrington R, Bunn J. Can maternal depression increase infant risk of illness and growth impairment in developing countries? Child. 2002 Jan;28(1):51–6. 10.1046/j.1365-2214.2002.00239.x 11856187

[48] Tomlinson M, Rabie S, Skeen S, Hunt X, Murray L, Cooper PJ. Improving mother–infant interaction during infant feeding: A randomised controlled trial in a low‐income community in South Africa. Infant Mental Health Journal. 2020 Nov;41(6):850–8. 32667053 10.1002/imhj.21881

[49] WHO. Infant feeding for the prevention of mother-to-child transmission of HIV [Internet]. 2023 [cited 2024 Aug 13]. Available from: https://www.who.int/tools/elena/interventions/hiv-infant-feeding

[50] CDC. Breastfeeding Recommendations and Benefits [Internet]. Centers for Disease Control and Prevention. 2022 [cited 2024 Aug 13]. Available from: https://www.cdc.gov/nutrition/infantandtoddlernutrition/breastfeeding/recommendations-benefits.html

[51] Rivi V, Petrilli G, Blom JMC. Mind the Mother When Considering Breastfeeding. Front Glob Womens Health. 2020 Sep 15;1:3. 34816148 10.3389/fgwh.2020.00003 PMC8593947

[52] Brown A, Rance J, Bennett P. Understanding the relationship between breastfeeding and postnatal depression: the role of pain and physical difficulties. J Adv Nurs. 2016 Feb;72(2):273–82. 26494433 10.1111/jan.12832 PMC4738467

[53] Mabetha K, Soepnel LM, Klingberg S, Mabena G, Motlhatlhedi M, Norris SA, Young women’s social support networks during pregnancy in Soweto, South Africa. African Journal of Primary Health Care & Family Medicine [Internet]. 2024 Apr 29 [cited 2024 Aug 14];16(1). Available from: http://www.phcfm.org/index.php/PHCFM/article/view/4146 10.4102/phcfm.v16i1.4146 PMC11079395 38708725

[54] Stanton AM, O’Cleirigh C, Knight L, Davey DLJ, Myer L, Joska JA, The importance of assessing and addressing mental health barriers to PrEP use during pregnancy and postpartum in sub‐Saharan Africa: state of the science and research priorities. J Int AIDS Soc [Internet]. 2022 Oct [cited 2022 Nov 8];25(10). Available from: https://onlinelibrary.wiley.com/doi/10.1002/jia2.26026 10.1002/jia2.26026 PMC9575939 36251124

[55] Republic of South Africa Department of Health. National Mental Health Policy Framework and Strategic Plan 2023–2030. 2023.

[56] Cornick R, Picken S, Wattrus C, Awotiwon A, Carkeek E, Hannington J, The Practical Approach to Care Kit (PACK) guide: developing a clinical decision support tool to simplify, standardise and strengthen primary healthcare delivery. BMJ Glob Health. 2018 Oct;3(Suppl 5):e000962. 10.1136/bmjgh-2018-000962 PMC6195147 30364419

[57] Nolvi S, Merz EC, Kataja EL, Parsons CE. Prenatal Stress and the Developing Brain: Postnatal Environments Promoting Resilience. Biological Psychiatry. 2023 May;93(10):942–52. 36870895 10.1016/j.biopsych.2022.11.023

[58] Abrahams Z, Boisits S, Schneider M, Honikman S, Lund C. Facilitators and barriers to detection and treatment of depression, anxiety and experiences of domestic violence in pregnant women. Sci Rep. 2023 Aug 1;13(1):12457. 37528133 10.1038/s41598-023-36150-z PMC10394005

