# Supplementary material for: Exploring the effects of mental health on bonding and caregiving among pregnant and postpartum persons with likely depression and/or PTSD in South Africa: A qualitative analysis
Source: Res Sq. 2024 Oct 24:rs.3.rs-5041479. Preprint. [Version 1] doi: 10.21203/rs.3.rs-5041479/v1 (PMC11537343; doi:10.21203/rs.3.rs-5041479/v1)
Supplement: Supplement 1 [file NIHPPRS5041479V1-supplement-1.pdf]

Supplementary Files

This is a list of supplementary files associated with this preprint. Click to download.

- Additionalfile1.pdf
